# *catena*-Poly[[[[3-(2-pyrid­yl)-1*H*-pyrazole]cadmium(II)]-μ-oxalato] dihydrate]

**DOI:** 10.1107/S1600536809043566

**Published:** 2009-10-28

**Authors:** Ling Zhu, Zhe An

**Affiliations:** a School of Chemistry and Life Science, Maoming University, Maoming 525000, People’s Republic of China

## Abstract

In the title compound, {[Cd(C_2_O_4_)(C_8_H_7_N_3_)]·2H_2_O}_*n*_, the Cd^II^ ion is chelated by two *O*,*O*′-bidentate oxalate ions and an *N*,*N*′-bidentate 3-(2-pyrid­yl)-1*H*-pyrazole mol­ecule, thereby generating a distorted *cis*-CdN_2_O_4_ octa­hedral geometry. Adjacent pairs of Cd ions are bridged by oxalate ions, resulting in wave-like polymeric chains propagating in [100]. The packing is consolidated by N—H—O and O—H—O hydrogen bonds.

## Related literature

For coordination compounds with pyridyl-pyrazolide ligands, see: Ward *et al.* (1998 ▶, 2001 ▶). 
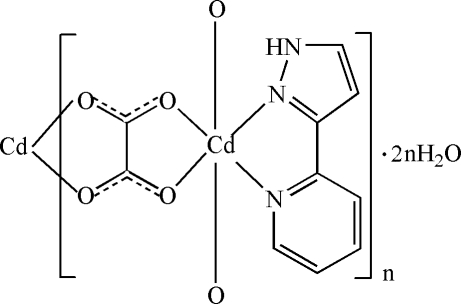


## Experimental

### 

#### Crystal data

[Cd(C_2_O_4_)(C_8_H_7_N_3_)]·2H_2_O
*M* *_r_* = 381.63
Triclinic, 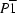

*a* = 7.920 (2) Å
*b* = 9.663 (2) Å
*c* = 9.675 (2) Å
α = 92.940 (4)°
β = 108.555 (3)°
γ = 106.164 (4)°
*V* = 666.2 (3) Å^3^
*Z* = 2
Mo *K*α radiation
μ = 1.67 mm^−1^
*T* = 293 K
0.12 × 0.10 × 0.08 mm

#### Data collection

Bruker SMART CCD diffractometer
Absorption correction: multi-scan (*SADABS*; Bruker, 2003 ▶) *T*_min_ = 0.825, *T*_max_ = 0.878
3416 measured reflections
2346 independent reflections
2247 reflections with *I* > 2σ(*I*)
*R*_int_ = 0.008

#### Refinement

*R*[*F* ^2^ > 2σ(*F* ^2^)] = 0.018
*wR*(*F* ^2^) = 0.050
*S* = 1.00
2346 reflections
193 parameters
6 restraints
H atoms treated by a mixture of independent and constrained refinement
Δρ_max_ = 0.38 e Å^−3^
Δρ_min_ = −0.31 e Å^−3^

### 

Data collection: *SMART* (Bruker, 2003 ▶); cell refinement: *SAINT-Plus* (Bruker, 2003 ▶); data reduction: *SAINT-Plus*; program(s) used to solve structure: *SHELXTL* (Sheldrick, 2008 ▶); program(s) used to refine structure: *SHELXTL*; molecular graphics: *SHELXTL*; software used to prepare material for publication: *SHELXTL*.

## Supplementary Material

Crystal structure: contains datablocks I, global. DOI: 10.1107/S1600536809043566/hb5161sup1.cif

Structure factors: contains datablocks I. DOI: 10.1107/S1600536809043566/hb5161Isup2.hkl

Additional supplementary materials:  crystallographic information; 3D view; checkCIF report

## Figures and Tables

**Table 1:** Selected bond lengths (Å)

Cd1—O1	2.2802 (16)
Cd1—O2^i^	2.2850 (17)
Cd1—O3	2.3286 (17)
Cd1—O4^ii^	2.3010 (16)
Cd1—N1	2.365 (2)
Cd1—N2	2.292 (2)

Symmetry codes: (i) 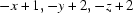; (ii) 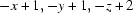.

**Table 2:** Hydrogen-bond geometry (Å, °)

*D*—H⋯*A*	*D*—H	H⋯*A*	*D*⋯*A*	*D*—H⋯*A*
N3—H3*A*⋯O5^iii^	0.86	1.85	2.696 (3)	169
O5—H2*W*⋯O2^iv^	0.82 (2)	2.20 (2)	2.861 (3)	138 (3)
O6—H3*W*⋯O4^v^	0.82 (4)	2.34 (3)	2.878 (3)	124 (3)
O6—H4*W*⋯O3^vi^	0.82 (4)	2.01 (4)	2.832 (3)	171 (4)

Symmetry codes: (iii) 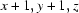; (iv) 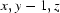; (v) 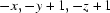; (vi) 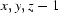.

**Fig. 1.**
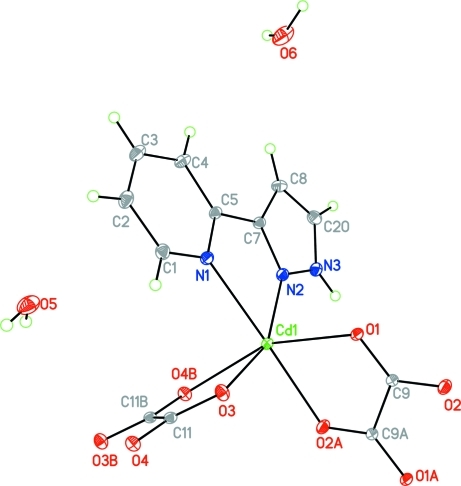
, A view of the title compound with the unique atom-labelling scheme. Displacement ellipsoids are drawn at the 30% probability level. Atoms labeled with A are at the symmetry position (-x,-y + 2,-z + 2).

**Fig. 2.**
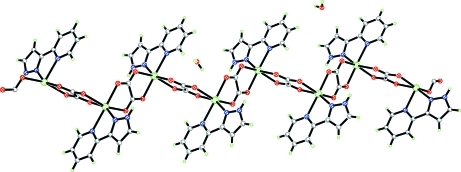
, A view of the chain strcuture of (I).

**Fig. 3.**
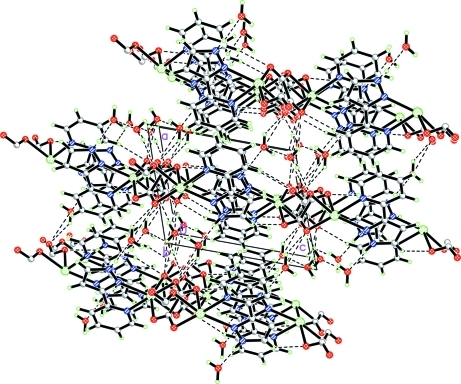
, A view of the packing strcuture of (I).

## supplementary crystallographic information

### Comment

Tridentate ligand 3-(2-pyridyl)pyrazole and its derivatives have been used widely in the construction of supramolecular architectures by way of metal-organic coordination (Ward *et al.* 1998; 2001).

As a continuation of these studies, we now report the crystal structure of the title complex.

As shown in figure 1, the Cd^II^ ions are hexcoordianted, chelated by two oxalate and one 3-(2-pyridyl)pyrazole ligand (Table 1). While each oxalate ligand acts as one bridige to chalate two Cd ions, forming one wave-like line with Cd···Cd distance being 5.950 /%A, shown in Figure 2. The structure is consolidated by N—H···O and O—H···O hydrogen bonds (Table 2, Figure 3).

### Experimental

A mixture of Cd(CH_3_COO)_2_.2H_2_O (1 mmol, 0.027 g), oxalic acid (1 mmol, 0.09 g), sodium hydroxide (0.04 g, 1 mmol) and 3-(2-pyridyl)pyrazole (1 mmol, 0.15 g) and water (12 ml) was stirred for 30 min in air. The mixture was then transferred to a 25 ml Teflon-lined hydrothermal bomb. The bomb was kept at 433 K for 72 h under autogenous pressure. Upon cooling, colorless prisms of (I) were obtained from the reaction mixture.

### Refinement

All hydrogen atoms bound to carbon were refined using a riding model with C—H = 0.93 and *U*_iso_(H) = 1.2U_eq_(C). Two solvent water molecules are refined by using the '*DFIX*' command with the hydrogen atoms were separated with 1.38 Å, and the lengths of bond H—O were constrained with 0.82 Å with error 0.02Å and *U*_iso_ = 1.5*U*_eq_ (O).

### Crystal data

**Table:**

[Cd(C_2_O_4_)(C_8_H_7_N_3_)]·2H_2_O	*Z* = 2
*M**_r_* = 381.63	*F*(000) = 376
Triclinic, *P*1	*D*_x_ = 1.902 Mg m^−^^3^
Hall symbol: -P 1	Mo *K*α radiation, λ = 0.71073 Å
*a* = 7.920 (2) Å	Cell parameters from 3167 reflections
*b* = 9.663 (2) Å	θ = 2.9–28.3°
*c* = 9.675 (2) Å	µ = 1.67 mm^−^^1^
α = 92.940 (4)°	*T* = 293 K
β = 108.555 (3)°	Block, colorless
γ = 106.164 (4)°	0.12 × 0.10 × 0.08 mm
*V* = 666.2 (3) Å^3^	

### Data collection

**Table:**

Bruker SMART CCD diffractometer	2346 independent reflections
Radiation source: fine-focus sealed tube	2247 reflections with *I* > 2σ(*I*)
graphite	*R*_int_ = 0.008
ω scans	θ_max_ = 25.0°, θ_min_ = 2.3°
Absorption correction: multi-scan (*SADABS*; Bruker, 2003)	*h* = −8→9
*T*_min_ = 0.825, *T*_max_ = 0.878	*k* = −11→9
3416 measured reflections	*l* = −11→10

### Refinement

**Table:**

Refinement on *F*^2^	Primary atom site location: structure-invariant direct methods
Least-squares matrix: full	Secondary atom site location: difference Fourier map
*R*[*F*^2^ > 2σ(*F*^2^)] = 0.018	Hydrogen site location: inferred from neighbouring sites
*wR*(*F*^2^) = 0.050	H atoms treated by a mixture of independent and constrained refinement
*S* = 1.00	*w* = 1/[σ^2^(*F*_o_^2^) + (0.031*P*)^2^ + 0.429*P*] where *P* = (*F*_o_^2^ + 2*F*_c_^2^)/3
2346 reflections	(Δ/σ)_max_ = 0.001
193 parameters	Δρ_max_ = 0.38 e Å^−^^3^
6 restraints	Δρ_min_ = −0.30 e Å^−^^3^

### Special details

**Table:**

Geometry. All e.s.d.'s (except the e.s.d. in the dihedral angle between two l.s. planes) are estimated using the full covariance matrix. The cell e.s.d.'s are taken into account individually in the estimation of e.s.d.'s in distances, angles and torsion angles; correlations between e.s.d.'s in cell parameters are only used when they are defined by crystal symmetry. An approximate (isotropic) treatment of cell e.s.d.'s is used for estimating e.s.d.'s involving l.s. planes.
Refinement. Refinement of *F*^2^ against ALL reflections. The weighted *R*-factor *wR* and goodness of fit *S* are based on *F*^2^, conventional *R*-factors *R* are based on *F*, with *F* set to zero for negative *F*^2^. The threshold expression of *F*^2^ > σ(*F*^2^) is used only for calculating *R*-factors(gt) *etc*. and is not relevant to the choice of reflections for refinement. *R*-factors based on *F*^2^ are statistically about twice as large as those based on *F*, and *R*- factors based on ALL data will be even larger.

### Fractional atomic coordinates and isotropic or equivalent isotropic displacement parameters (Å^2^)

**Table:**

	*x*	*y*	*z*	*U*_iso_*/*U*_eq_	
Cd1	0.49223 (2)	0.741369 (17)	0.821503 (17)	0.03309 (8)	
C1	0.1758 (4)	0.4289 (3)	0.6244 (3)	0.0458 (6)	
H1	0.1605	0.4120	0.7141	0.055*	
C2	0.0730 (4)	0.3235 (3)	0.5038 (4)	0.0543 (7)	
H2	−0.0085	0.2366	0.5120	0.065*	
C3	0.0924 (4)	0.3483 (3)	0.3708 (3)	0.0531 (7)	
H3	0.0232	0.2791	0.2869	0.064*	
C4	0.2164 (4)	0.4777 (3)	0.3633 (3)	0.0462 (6)	
H4	0.2317	0.4967	0.2741	0.055*	
C5	0.3179 (3)	0.5791 (3)	0.4901 (3)	0.0339 (5)	
C7	0.4569 (3)	0.7167 (3)	0.4902 (3)	0.0342 (5)	
C8	0.4998 (4)	0.7780 (3)	0.3728 (3)	0.0475 (6)	
H8	0.4419	0.7414	0.2727	0.057*	
C9	0.4002 (3)	0.9947 (2)	0.9452 (2)	0.0311 (5)	
C11	0.4082 (3)	0.5038 (2)	1.0129 (2)	0.0295 (5)	
C20	0.6454 (4)	0.9036 (3)	0.4371 (3)	0.0496 (7)	
H20	0.7065	0.9693	0.3884	0.060*	
N1	0.2973 (3)	0.5554 (2)	0.6200 (2)	0.0365 (4)	
N2	0.5697 (3)	0.8005 (2)	0.6184 (2)	0.0351 (4)	
N3	0.6837 (3)	0.9143 (2)	0.5838 (2)	0.0421 (5)	
H3A	0.7701	0.9847	0.6474	0.050*	
O1	0.3288 (2)	0.89932 (18)	0.83387 (18)	0.0377 (4)	
O2	0.3257 (2)	1.0843 (2)	0.9777 (2)	0.0450 (4)	
O3	0.3446 (2)	0.60271 (19)	0.9645 (2)	0.0409 (4)	
O4	0.3377 (2)	0.41014 (18)	1.07962 (18)	0.0364 (4)	
O5	−0.0175 (3)	0.1137 (3)	0.7858 (3)	0.0753 (7)	
H1W	−0.063 (4)	0.067 (5)	0.841 (4)	0.113*	
H2W	0.0949 (15)	0.126 (5)	0.805 (4)	0.113*	
O6	0.0648 (3)	0.6774 (3)	0.0467 (3)	0.0863 (9)	
H3W	−0.016 (4)	0.703 (5)	−0.013 (4)	0.130*	
H4W	0.143 (5)	0.660 (5)	0.015 (4)	0.130*	

### Atomic displacement parameters (Å^2^)

**Table:**

	*U*^11^	*U*^22^	*U*^33^	*U*^12^	*U*^13^	*U*^23^
Cd1	0.04230 (12)	0.02946 (11)	0.02728 (11)	0.01333 (8)	0.01047 (8)	0.00063 (7)
C1	0.0421 (14)	0.0385 (14)	0.0520 (16)	0.0092 (11)	0.0127 (12)	0.0075 (12)
C2	0.0399 (14)	0.0388 (14)	0.071 (2)	0.0056 (11)	0.0093 (14)	−0.0040 (13)
C3	0.0412 (15)	0.0483 (16)	0.0574 (17)	0.0102 (12)	0.0071 (13)	−0.0147 (13)
C4	0.0400 (14)	0.0520 (16)	0.0393 (14)	0.0122 (12)	0.0092 (11)	−0.0124 (12)
C5	0.0319 (12)	0.0368 (12)	0.0333 (12)	0.0144 (10)	0.0095 (9)	−0.0023 (10)
C7	0.0338 (12)	0.0372 (12)	0.0312 (12)	0.0138 (10)	0.0093 (10)	0.0004 (10)
C8	0.0505 (16)	0.0583 (17)	0.0315 (13)	0.0162 (13)	0.0121 (11)	0.0055 (12)
C9	0.0329 (12)	0.0288 (11)	0.0286 (11)	0.0096 (9)	0.0070 (10)	0.0024 (9)
C11	0.0301 (11)	0.0357 (12)	0.0242 (10)	0.0137 (9)	0.0091 (9)	0.0009 (9)
C20	0.0571 (17)	0.0508 (16)	0.0447 (15)	0.0159 (13)	0.0220 (13)	0.0172 (13)
N1	0.0355 (10)	0.0348 (10)	0.0362 (11)	0.0109 (8)	0.0090 (9)	0.0011 (8)
N2	0.0384 (11)	0.0332 (10)	0.0336 (10)	0.0108 (8)	0.0132 (9)	0.0027 (8)
N3	0.0458 (12)	0.0328 (11)	0.0432 (12)	0.0079 (9)	0.0138 (10)	0.0036 (9)
O1	0.0402 (9)	0.0347 (9)	0.0311 (8)	0.0152 (7)	0.0014 (7)	−0.0049 (7)
O2	0.0393 (9)	0.0438 (10)	0.0444 (10)	0.0208 (8)	0.0005 (8)	−0.0119 (8)
O3	0.0448 (10)	0.0459 (10)	0.0487 (10)	0.0268 (8)	0.0260 (8)	0.0158 (8)
O4	0.0361 (9)	0.0426 (9)	0.0392 (9)	0.0170 (7)	0.0197 (7)	0.0123 (7)
O5	0.0519 (13)	0.0818 (18)	0.0707 (16)	0.0203 (13)	−0.0022 (11)	−0.0180 (13)
O6	0.0464 (13)	0.103 (2)	0.0975 (19)	0.0238 (13)	0.0171 (13)	−0.0409 (16)

### Geometric parameters (Å, °)

**Table:**

Cd1—O1	2.2802 (16)	C8—C20	1.370 (4)
Cd1—O2^i^	2.2850 (17)	C8—H8	0.9300
Cd1—O3	2.3286 (17)	C9—O1	1.245 (3)
Cd1—O4^ii^	2.3010 (16)	C9—O2	1.253 (3)
Cd1—N1	2.365 (2)	C9—C9^i^	1.571 (4)
Cd1—N2	2.292 (2)	C11—O3	1.245 (3)
C1—N1	1.341 (3)	C11—O4	1.247 (3)
C1—C2	1.369 (4)	C11—C11^ii^	1.572 (4)
C1—H1	0.9300	C20—N3	1.345 (4)
C2—C3	1.369 (5)	C20—H20	0.9300
C2—H2	0.9300	N2—N3	1.346 (3)
C3—C4	1.380 (4)	N3—H3A	0.8600
C3—H3	0.9300	O2—Cd1^i^	2.2850 (17)
C4—C5	1.386 (3)	O4—Cd1^ii^	2.3010 (16)
C4—H4	0.9300	O5—H1W	0.82 (4)
C5—N1	1.341 (3)	O5—H2W	0.82 (2)
C5—C7	1.471 (3)	O6—H3W	0.82 (4)
C7—N2	1.334 (3)	O6—H4W	0.82 (4)
C7—C8	1.400 (4)		
			
O1—Cd1—O2^i^	73.10 (6)	N2—C7—C8	110.4 (2)
O1—Cd1—N2	99.85 (7)	N2—C7—C5	119.4 (2)
O2^i^—Cd1—N2	110.73 (7)	C8—C7—C5	130.2 (2)
O1—Cd1—O4^ii^	153.29 (6)	C20—C8—C7	105.1 (2)
O2^i^—Cd1—O4^ii^	89.10 (6)	C20—C8—H8	127.5
N2—Cd1—O4^ii^	105.11 (6)	C7—C8—H8	127.5
O1—Cd1—O3	88.25 (6)	O1—C9—O2	125.3 (2)
O2^i^—Cd1—O3	90.72 (7)	O1—C9—C9^i^	118.1 (2)
N2—Cd1—O3	158.43 (7)	O2—C9—C9^i^	116.6 (2)
O4^ii^—Cd1—O3	71.86 (6)	O3—C11—O4	125.3 (2)
O1—Cd1—N1	106.71 (6)	O3—C11—C11^ii^	117.3 (2)
O2^i^—Cd1—N1	177.56 (7)	O4—C11—C11^ii^	117.4 (2)
N2—Cd1—N1	71.72 (7)	N3—C20—C8	107.4 (2)
O4^ii^—Cd1—N1	90.22 (7)	N3—C20—H20	126.3
O3—Cd1—N1	86.84 (7)	C8—C20—H20	126.3
N1—C1—C2	123.5 (3)	C1—N1—C5	117.8 (2)
N1—C1—H1	118.3	C1—N1—Cd1	126.77 (18)
C2—C1—H1	118.3	C5—N1—Cd1	115.34 (15)
C3—C2—C1	118.7 (3)	C7—N2—N3	105.76 (19)
C3—C2—H2	120.7	C7—N2—Cd1	116.09 (15)
C1—C2—H2	120.7	N3—N2—Cd1	137.04 (15)
C2—C3—C4	119.0 (3)	C20—N3—N2	111.3 (2)
C2—C3—H3	120.5	C20—N3—H3A	124.3
C4—C3—H3	120.5	N2—N3—H3A	124.3
C3—C4—C5	119.4 (3)	C9—O1—Cd1	115.63 (14)
C3—C4—H4	120.3	C9—O2—Cd1^i^	115.99 (14)
C5—C4—H4	120.3	C11—O3—Cd1	115.95 (14)
N1—C5—C4	121.6 (2)	C11—O4—Cd1^ii^	116.68 (14)
N1—C5—C7	116.4 (2)	H1W—O5—H2W	115 (4)
C4—C5—C7	121.9 (2)	H3W—O6—H4W	115 (4)

Symmetry codes: (i) −*x*+1, −*y*+2, −*z*+2; (ii) −*x*+1, −*y*+1, −*z*+2.

### Hydrogen-bond geometry (Å, °)

**Table:**

*D*—H···*A*	*D*—H	H···*A*	*D*···*A*	*D*—H···*A*
N3—H3A···O5^iii^	0.86	1.85	2.696 (3)	169
O5—H2W···O2^iv^	0.82 (2)	2.20 (2)	2.861 (3)	138 (3)
O6—H3W···O4^v^	0.82 (4)	2.34 (3)	2.878 (3)	124 (3)
O6—H4W···O3^vi^	0.82 (4)	2.01 (4)	2.832 (3)	171 (4)

Symmetry codes: (iii) *x*+1, *y*+1, *z*; (iv) *x*, *y*−1, *z*; (v) −*x*, −*y*+1, −*z*+1; (vi) *x*, *y*, *z*−1.
